# Preliminary study: Health and performance assessment in broiler chicks following application of six different hatching egg disinfection protocols

**DOI:** 10.1371/journal.pone.0232825

**Published:** 2020-05-14

**Authors:** Wiebke Tebrün, Gerzon Motola, Mohamed Hafez Hafez, Josef Bachmeier, Volker Schmidt, Kevin Renfert, Christian Reichelt, Sarah Brüggemann-Schwarze, Michael Pees

**Affiliations:** 1 University Teaching Hospital, Department for Birds and Reptiles, University of Leipzig, An den Tierkliniken, Leipzig, Germany; 2 Institute of Poultry Diseases, Freie Universität Berlin, Königsweg, Berlin, Germany; 3 Brüterei Süd, Regenstauf, Germany; Tokat Gaziosmanpasa University, TURKEY

## Abstract

As part of a Germany-wide project that evaluates strategies for the reduction of multi-resistant bacteria along the poultry production chain, the impact of different hatching egg disinfectants on hatchability and health of the broiler chicks was evaluated. Animal trials were conducted with extended-spectrum beta-lactamase- (**ESBL**) producing *Escherichia (****E*.***) coli* contaminated hatching eggs and six disinfection protocols that used formaldehyde, hydrogen peroxide, low-energy electron irradiation, peracetic acid and an essential oil preparation. Each protocol was tested on a group of 50 chicks. Equally sized positive and negative control groups were carried along for each trial. Hatchability, mortality and body weight were recorded as performance parameters. During necropsy of half of the animals in each group on day 7 and 14 respectively, macroscopic abnormalities, body weight, weights of liver and gut convolute were recorded and a range of tissue samples for histological examination were collected as part of the health assessment. A decrease in hatchability was recorded for spray application of essential oils. Body weight development was overall comparable, in several groups even superior, to the Ross308 performance objectives, but a reduced performance was seen in the hydrogen peroxide group. Histologically, lymphoid follicles were regularly seen in all sampled organs and no consistent differences were observed between contaminated and non-contaminated groups. Significances were infrequently and inconsistently seen. In conclusion, remarkable findings were a decrease in hatchability caused by the essential oils spray application and a reduced body weight development in the hydrogen peroxide group. Therefore, the essential oils preparation as spray application was deemed inappropriate in practice, while the application of hydrogen peroxide was considered in need of further research. The other trial results indicate that the tested hatching egg disinfectants present a possible alternative to formaldehyde.

## Introduction

Hatching egg disinfection is a vital method for the reduction of the bacterial load in the poultry production chain, preventing problems with hatchability and chick performance.[1] Formaldehyde is commonly used in hatcheries for hatching egg disinfection as it is inexpensive, easy to apply and effective.[2, 3] However, it irritates the upper respiratory tract [4, 5] and is teratogenic and carcinogenic [6], making its use hazardous for personel and chicks. In 2014, the European Union officially reclassified formaldehyde as carcinogenic, mutagenic and acutely toxic (EU regulation 605/2014) and in Germany the use of formaldehyde in its gaseous form is restricted to certified users under certain technical parameters (GMBl 2013). A potential withdrawal from the market has already been discussed since the 1980’s.[7] Apart from formaldehyde, various other disinfectants have been tested for use on hatching eggs.

Hydrogen peroxide has generally demonstrated good bactericidal efficacy without adverse effects on hatchability and chick quality, in some degree depending on the method of application and concentration of the product.[8–14] Essential oils, specifically wetting the eggs with an oregano oil and alcohol mixture, have been tested on quail eggs [15] resulting in lower bacterial count on the egg shell than formaldehyde and lower mortality. The antimicrobial activity is attributed to reactive OH-groups of the oregano oil.

The use of colloidal silver as an egg disinfectant lead to a reduction of the bacterial load and better broiler performance than a control group treated with formaldehyde [16], but bears the risk of silver residues in the hatching chicks.[17] Colloidal silver works by interacting with sulfurous amino acids and DNA.[16] Electrolyzed water, with its low pH, high oxidation reduction potential and free chlorine, showed good efficacy against pathogens and indicator bacteria, with the advantage of being nontoxic.[18] Castaneda et al. [19] tried electron irradiation at 1 kGy and 2 kGy on hatching eggs with no recorded impact on hatchability or broiler performance and which lead to a reduction of colony forming units of *Salmonella* Enteritidis. Phenolic substances were applied with no negative impact on hatchability or chick quality [20], however the antimicrobial efficacy of the treatment was not tested. The use of peracetic acid on hatching eggs lead to a significant reduction of aerobic bacteria and a significant reduction of Enterobacteriaceae and was suggested as possible alternative for formaldehyde hatching egg disinfection. [21]

UV light was tested alone [22] and in combination with hydrogen peroxide [23] with a favorable effect on bacterial contamination due to advanced oxidation processes resulting in the production of highly reactive oxygen species.[23] A disadvantage was heat development when application times exceeded 8 minutes.[12] Quaternary ammonium preparations appeared to decrease hatchability with increasing concentrations [20], but also did not successfully eliminate bacteria from the egg shell.[24, 11] When applying ozone, Whistler and Sheldon [7] noticed a bactericidal effect comparable to formaldehyde, but a negative impact on hatchability. However, other studies did not detect changes in hatchability with ozone.[25] Its bactericidal effect is due to its high oxidative potential and the production of radicals in a humid environment.[26]

It is important to note that egg disinfection alone does not help to prevent the occurrence of bacteria in the hatching environment. Transmission of bacteria to the hatchery via the egg surface has been tracked.[27] During the hatching process horizontal transmission inside the incubator contributes to the bacterial load.[9, 27] Cleaning and disinfection of hatchery facilities, with special regard to heating, ventilation and humidification plants, is therefore important in addition to hatching egg disinfection.

For some time, extended-spectrum beta-lactamase- (**ESBL**) producing *Escherichia (****E*.***) coli* have been reported as an emerging threat in poultry production. These bacteria have been isolated from poultry farms all over the world [28–30] and can be found at all stages in the broiler production chain.[31] Hatching eggs have been identified as carriers and a potential source.[32] There are findings of ESBL producing bacteria on meat [33] in retail stores and easy contamination of kitchen equipment and personnel has been illustrated.[34] However, it is still unclear whether ESBL producing *E*. *coli* from poultry meat cause illness in humans [35], as for example a majority of isolates from healthy poultry flocks lacked virulence genes associated with human-pathogenic strains.[36] Krizman et al. [37], on the other hand, found similar ESBL-producing *E*. *coli* strains in meat products and people with diarrhea. A Swedish study showed that 4.7% of the healthy population carry ESBL-producing *E*. *coli* strains with a low pathogenicity [38] as opposed to people with a blood stream infection that harbor highly pathogenic strains.

The aim of this study was therefore to evaluate the application of different hatching egg disinfectants on ESBL-producing *E*. *coli* contaminated hatching eggs and their impact on hatchability and health of the hatchlings over a period of 14 days. The study was conducted as part of a Germany-wide project that evaluates strategies for the reduction of multi-resistant bacteria along the poultry production chain (EsRAM, FUZ 28177 01714).

## Material and methods

### Disinfectants

The efficacy of five disinfectants using a total of six different application protocols was tested on hatching eggs before setting. Formaldehyde (Jäklechemie, Nürnberg, Germany) disinfection was selected as the reference method, as it is commonly used in hatcheries. A hydrogen peroxide preparation, Wessoclean® K50 Goldline (Bio-Clean B.V., Arnhem, Netherlands), was applied as an aerosol with a particle size of five to ten micrometers via Veugen-injector. An Evonta® EggClean prototype (Evonta, Dresden, Germany), using low-energy electron irradiation with energy levels of 200 keV and 60 kGy was tested. A peracetic acid solvent cage preparation, Wofasteril® (Kesla Hygiene AG, Bitterfeld-Wolfen, Germany), was foamed over the eggshell. Finally, an essential oil product, Vitasan Spray® (EW Nutrition, GmbH, Visbek, Germany), was applied using two different methods, via spray bottle and via ultrafogger nebulization. For details see Table 1.

**Table 1:** Disinfection methods and protocols used in this study.

Compound	Active substance	Application method	Concentration	Application protocol
**Formaldehyd Biozid 20% (Jäklechemie, Nürnberg, Germany)**	Formaldehyde	Fumigation	44 ml/m^3^	15 min exposure time, 10 min neutralization time with ammonia, 300 min ventilation
**Wessoclean® K50 Goldline (Bio-Clean B.V., Arnhem, Netherlands)**	Hydrogen peroxide/acid mix	Aerosol (particle size 5–10 μm)	Hydrogen peroxide: 0.5 ml/m^3^, ethanol: 500 ml/m^3^, propan-2-ol: 200 ml/m^3^	1 min spray, 50 min exposure time
**Evonta EggClean® Prototype (Evonta, Dresden, Germany)**	Low energy electron flow	Radiation	200 keV, 60 kGy	1 s radiation
**1+1 Wofasteril SC super (Kesla Hygiene AG, Bitterfeld-Wolfen, Germany)**	Peracetic acid in micro cages	Foam	0.5% (1 ml Wofasteril, 1 ml Alcapur, 198 ml distilled water)	60 min exposure time, followed by washing with PBS
**Vitasan® Spray (EW Nutrition, GmbH, Visbek, Germany)**	Essential oils	Spray	5% (5 ml product with 95 ml distilled water)	20 min exposure time
**Vitasan® Spray (EW Nutrition, GmbH, Visbek, Germany)**	Essential oils	Fogging	5% (5 ml product with 95 ml distilled water)	6 min fogging, 20 min exposure time

### Procedure

Based on biostatistical evaluation with body weight development as the primary factor, we chose a group size of 50 animals. For each disinfectant trial, we used 60 eggs per group of the same Ross 308 parent flock (Geflügelhof Möckern, Gommern, Germany). The identity of the parent flocks varied between the trials. However, to maintain consistent thickness and porosity of the eggshell between trials, eggs were only collected from parent flocks in their 14^th^ to 16^th^ week of laying. One test and one control group were contaminated for each trial. The eggs were warmed at 37°C for 2–3 hours. Different test groups were then immersed for 5 minutes in cold (4–6°C) bacterial suspension containing 10^8^ colony forming units (**CFU**)/ml of ESBL producing *E*. *coli* that was isolated from one day old chicks.(39) Contamination with the *E*. *coli* strain 10682, phylogroup B 1, enzyme-variant CTX-M-1 [39] was achieved via temperature difference egg-dipping.[40] The eggs were allowed to dry at room temperature for 30 minutes and transported to animal trial facilities.

Disinfectants were applied as listed above (see Table 1). Each disinfectant was tested alongside positive and negative control groups that were defined as follows: Group A was contaminated and disinfected, Group B was contaminated but not disinfected, and Group C was not contaminated and not disinfected. The experiments ran from July 2017 to August 2018, each trial blocking the facilities for 5 weeks followed by a service interval of at least 2 weeks.

Standard egg incubators (*Top Profi-120*, Co. Hemel, Verl, Germany) were used in separate rooms for each group. The rooms and incubators were cleaned, then disinfected using Safe Sept surface disinfection spray (Henry Schein, Langen, Germany), a disinfectant with quaternary ammonium compounds, with an exposure time of two hours. Before restocking the surfaces were microbiologically sampled to exclude a contamination with Enterobacteriaceae. The hatching eggs were incubated according to standard protocols at 37.8° C and 53% relative humidity from day 1 to day 17, and were turned every 3 hours. On day 18 the eggs were candled and the percentages of infertile eggs, dead embryos and fertile eggs were recorded. After removal of dead or non-fertile eggs, the hatching group size was adjusted to 50 eggs. The eggs were then transferred to hatcher baskets and returned to the same incubator, adjusting the settings to 37.6° C, 73% relative humidity and no egg turning until hatching. On day 21, the number of hatched chicks was recorded to calculate the hatching rate.

After hatching, the chicks were housed in enclosures of 4 m^2^ that were located in different rooms for each group and, in a different house for the not contaminated group C, with separate supply and exhaust air. Rooms and hygiene protocols were approved by authorities according to bio safety level 2 standards (EU directive 2000/54/EG). The animals were fed ad libitum with commercial feed (Hähnchen Starter, Mischfutter Werke, Mannheim, Germany). The food was offered in 3 round dishes of 23 cm diameter per enclosure, according to German Housing Regulations (207/43/EG, Tierschutz-Nutztierhaltungs-VO). The animals were checked twice daily until day 14, refilling the dishes when necessary to prevent fasting periods. They were weighed as a group on days 1, 7, and 14, as well as every second to third day in between. On day 7, half of the animals from each group were euthanized for necropsy using an overdose of isoflurane; the other half was euthanized and necropsied on day 14 using the same euthanasia technique. As the chicks were euthanized in a box using isoflurane, they stayed in an upright to lateral recumbent position. Exsanguination was not performed and all necropsies were performed within two hours after euthanasia.

Abandon criteria were defined, in case individual chicks showed signs of poor health (details on humane endpoint criteria are given in Tables 2 and 3). In case of sudden death, animals were necropsied and samples taken according to the usual necropsy protocol. The animal experiment was approved by the Landesdirektion Sachsen (trial application no. TVV 37/16).

**Table 2:** Humane endpoints–instructions to be followed according to scoring in Table 3.

Score	Instructions
A	Occurrence of one of the signs in category A: intensified observation, if necessary separation within the flock. Occurrence of more than one sign in category A: see instructions for category B
B	Occurrence of one of the signs in category B or more than one sign in category B: intensified observation (at least every three hours between 7 a.m. and 9 p.m.), in order to determine any intensification of the signs, if necessary separation within the flock. In case of combination of group B signs with ataxia, pain, paleness or bleeding, the animal is/the animals are to be euthanized humanely immediately (see category C).
C	Single or multiple animals are removed from the experimental setup and are euthanized humanely immediately using an overdose of isoflurane

**Table 3:** Humane endpoints–definition of criteria and scoring.

**Signs—individuals**	**Score**
Ruffled, untended plumage	A
Non-recurring sticky or soiled cloacal region	A
Mild lameness (weight is still put on the affected limb)	B
Distension of the abdomen	B
Mildly intensified respiration, increased respiratory rate or open-beak breathing	B
Mild/superficial lesion	B
Automutilation and/or loss of feathers	B
Sunken eyes	B
Severe lameness, no weight is put on the affected limb	C
Blood at body orifices, bloody feces	C
Moderately to severely intensified respiration, wheezing, other respiratory sounds	C
Longer lasting sticky or soiled cloacal region (over several observation intervals)	C
Seizures, ataxia, torticollis, opisthotonus	C
Apathy/moderately to severely reduced general condition (animal can no longer be mobilized, eyes stay closed upon approach/touching)	C
Abscesses	C
Visible malformations	C
Lesions with poor prognosis	C
**Signs—flock**	**Score**
Feces deviate in amount, colour and texture	A
Reduced or increased feed or water intake	A
Abnormal feces: occurrence of distinctly wet litter	A
Irregular distribution of animals in the pen (clustered in the centre or in the periphery)	A
Divergence in body weight: Occurrence of individuals with less than 50% of the mean body weight of the flock	B
Remarkable vocal expression	B
Cannibalism	C

To compare the three groups, performance parameters including hatching rate (in %), body weight development in comparison to a standard curve for Ross 308 (Ross 308 Performance Objectives, 2014) and mortality (in %) were calculated. Chicks that were euthanized according to abandon criteria were included in the mortality rate, whereas chicks that were euthanized because of congenital defects (day 1) were not included, but recorded. During necropsy, individual body weight, weight of liver and gut convolute were noted. For the pathological examination, criteria were defined to conduct a general organ assessment with special emphasis on the possible impact of a bacterial infection to the immune system, as well as possible side effects of the disinfection method on the organ development. Beside the general assessment following published standards [41,42,43], special emphasis was given to the criteria listed in Table 4.

**Table 4:** Definition of evaluation criteria for expected alterations in the post mortem examination on day 7 and 14. Criteria were scored as absent/present. Not all criteria were detected, and some were summed up for evaluation.

**Organ**	**Findings**	**Interpretation**
Macroscopy		
Air sacs, serosa	fibrinous deposits, increased vascularization	Indication for aerosacculitis/serositis [41]
	white, chalky deposits	urate deposition [41]
Lung	dirty-red discoloration, exsudate	Indication for pneumonia [44]
	reddening	Hypostasis due to euthanasia
Liver	Yellow discoloration and soft texture	Physiological on day 7, glycogen storage [45], at an advanced age indication for storage disease [41]
	Enlargement, brittle texture, discoloration	Indication for hepatitis, degeneration or storage disease [41]
Intestines	Intestinal wall thickened, discoloration	Indication for enteritis [41, 46]
Cloaca	Adhering faeces	Indication for maldigestion and dysbiosis [47]
	Reddening and swelling	Indication for inflammatory reaction [41]
Kidneys	Texture changes, enlargement, discoloration	Indication for nephritis or nephrosis [48]
	multifocal to diffuse white, crystal-like alterations	specific indication for urate deposition [41]
Yolk sac	Still present/larger than anticipated, wall thickened, with content	Yolk sac retention, indication for retarded development or low-grade non-specific inflammatory process [41, 49, 50, 51]
	Thickening of the wall, reddening or vascular infiltration, adhesion to inner organs, exudate	Yolk sac inflammation, specific sign for an inflammation caused by infection, commonly seen with coli granulomatosis [41, 44, 46]
**Alterations**	**Organ**	**Interpretation (references)**
Histology		
Cytoplasmic vacuolation	Liver, kidney, reticular cells of the spleen	Indication for degeneration or storage disease [52]
Active Kupffer cells	Liver	Indication for non-specific hepatic injury [52, 53]
(multi-)focal lymphoid follicles	Liver, lung, kidney, intestine	Antigenic stimulation, non-specific, incidentally, indication for inflammatory processes [52]
Depletion of lymphoid follicles	Spleen	Indication for toxic or infectious conditions [52, 54, 55]
Hyperplasia of lymph follicles	Spleen	Antigenic stimulation or disease [52]
Fibrin deposition, mixed population of heterophils, lymphocytes and histiocytes, necrosis, hemosiderophagocytosis	Spleen, liver, lung, kidney, intestine	Indication for bacterial infection [54, 56]
Extramedullary hematopoiesis	Liver, kidney	Non-specific, incidentally [57, 58]

**Fig 1:**
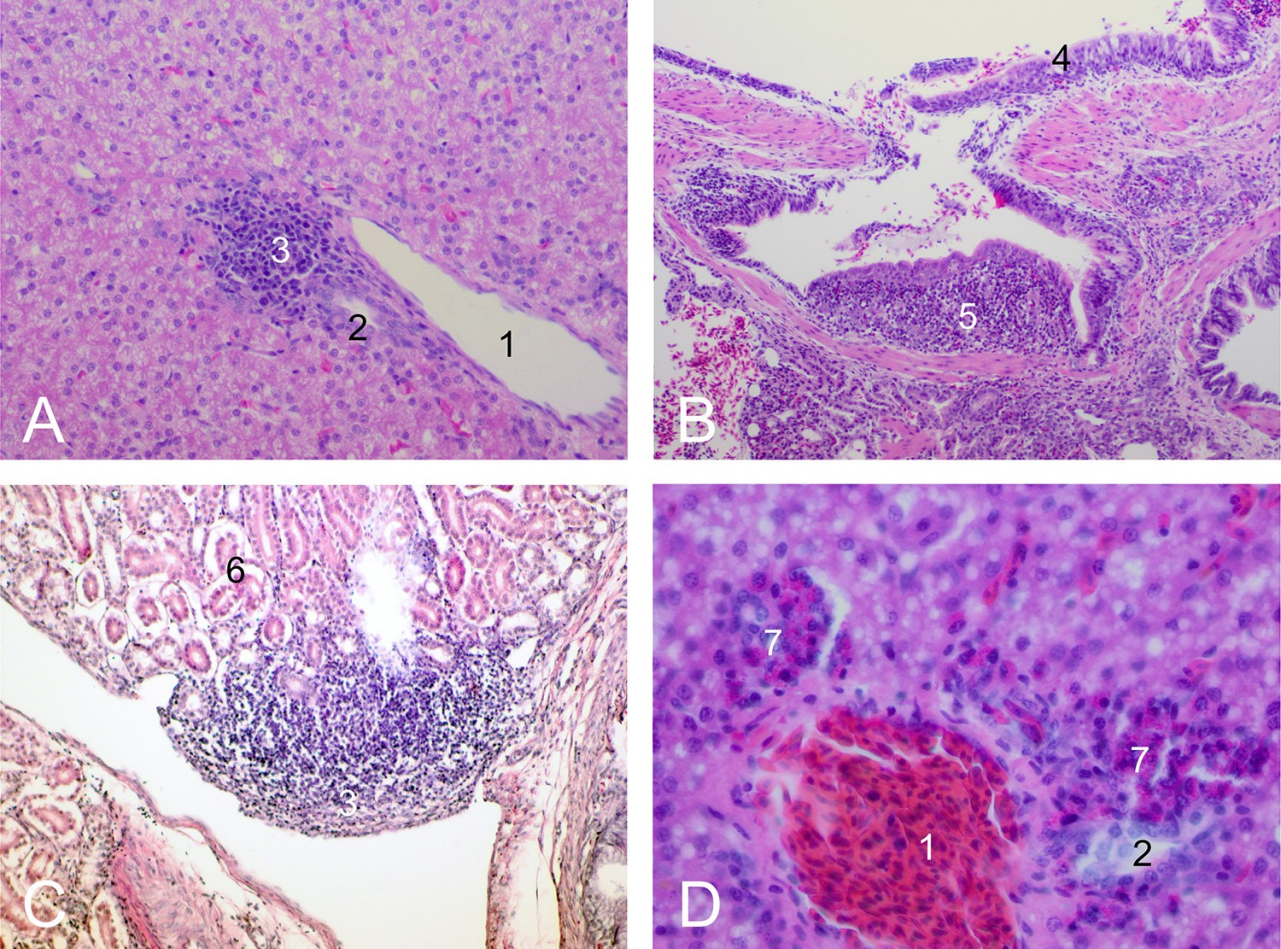
Tissue sections, light microscopy, H&E stain, A,C: 200x magnification, B: 100x magnification, D: 400x magnification, dissection day 7 A: liver tissue, lymphoid follicles B: lung tissue, lymphoid follicles C: kidney tissue, lymphoid follicles D: liver tissue, extramedullary hematopoiesis (1—portal vessel, 2 –bile duct, 3 –perivascular lymphoid follicles, 4 –lung epithelium, 5, periparabronchial lymphoid follicles, 6 –tubules, 7 –extramedullary hematopoiesis).

Special emphasis was given to the consequences of an *E*. *coli* infection as well as the potential toxic effects of the disinfection period on the organ development. Special attention was paid to the omphalic region and the yolk sac, as omphalitis is an important disease condition in chicks and commonly caused by *E*. *coli* infections [41]. A yellow discoloration and soft texture of the liver during necropsy on day 7 was still deemed physiological in chicks due to mobilization of fatty acids and cholesterol from the yolk sac.[45] Yolk sac alterations included yolk sac retention and yolk sac inflammation. As both pathological findings are etiologically related [49] and could not always be clearly differentiated with the criteria we used (Table 4), they were summed up under the term "abnormal" for the statistical evaluation. Abnormal findings of the kidneys were unspecific and consisted of brittle texture and pale colour. To measure the gut weight, the guts were excised proximally at the duodenal-gizzard junction, distally directly at the cloaca and were weighed as a whole, including the ingesta. Tissue samples from liver, lung, duodenum, spleen and kidney were fixed in 4% formaldehyde, then dehydrated, embedded in paraffin wax, and sectioned at 4 μm thickness, mounted on glass slides and stained with hematoxylin and eosin (Laboratory Protocols, Veterinary Pathology, University of Bristol, UK, http://www.bris.ac.uk/Depts/PathAndMicro/cpl/lablinks.html) for examination by light microscopy. The organ tissues were assessed following reported evaluation standards [52] and the criteria we defined (Table 4). Special attention was paid to heterophilic infiltrations, lymphoid follicles, extramedullary hematopoiesis, depletion of lymph follicles in the spleen and organ degeneration. Common findings are given in Fig 1. The histological assessment was conducted as a two-stage scoring, determining presence or absence of those lesions. To assess the overall health status of the respective groups, mean body weight and mean relative liver and gut weight (in %) were calculated.

### Statistics

Statistical evaluation between all groups in each single trial was done using the program SPSS 22.0 (SPSS, IBM, Armonk, USA). Potential differences between the different disinfectant trials were expected to be not meaningful due to seasonal effects and differences in the identities of the parent flocks, they were therefore not calculated. Based on data evaluation, a standard distribution was ascertained for the majority of the metrical parameters, but not for all of them. Therefore, the Mann-Whitney-U-test was used to identify significant relations concerning body, liver and gut weight with significance defined at p ≤ 0.05. Body weights that were recorded every second to third day as a group were not statistically evaluated. The frequency of macroscopic abnormalities of yolk sacs and kidneys, as well as histologic findings in liver, lungs and kidneys were evaluated using Fisher’s exact test, due to group sizes with n ≤ 20.

## Results

**Fig 2:**
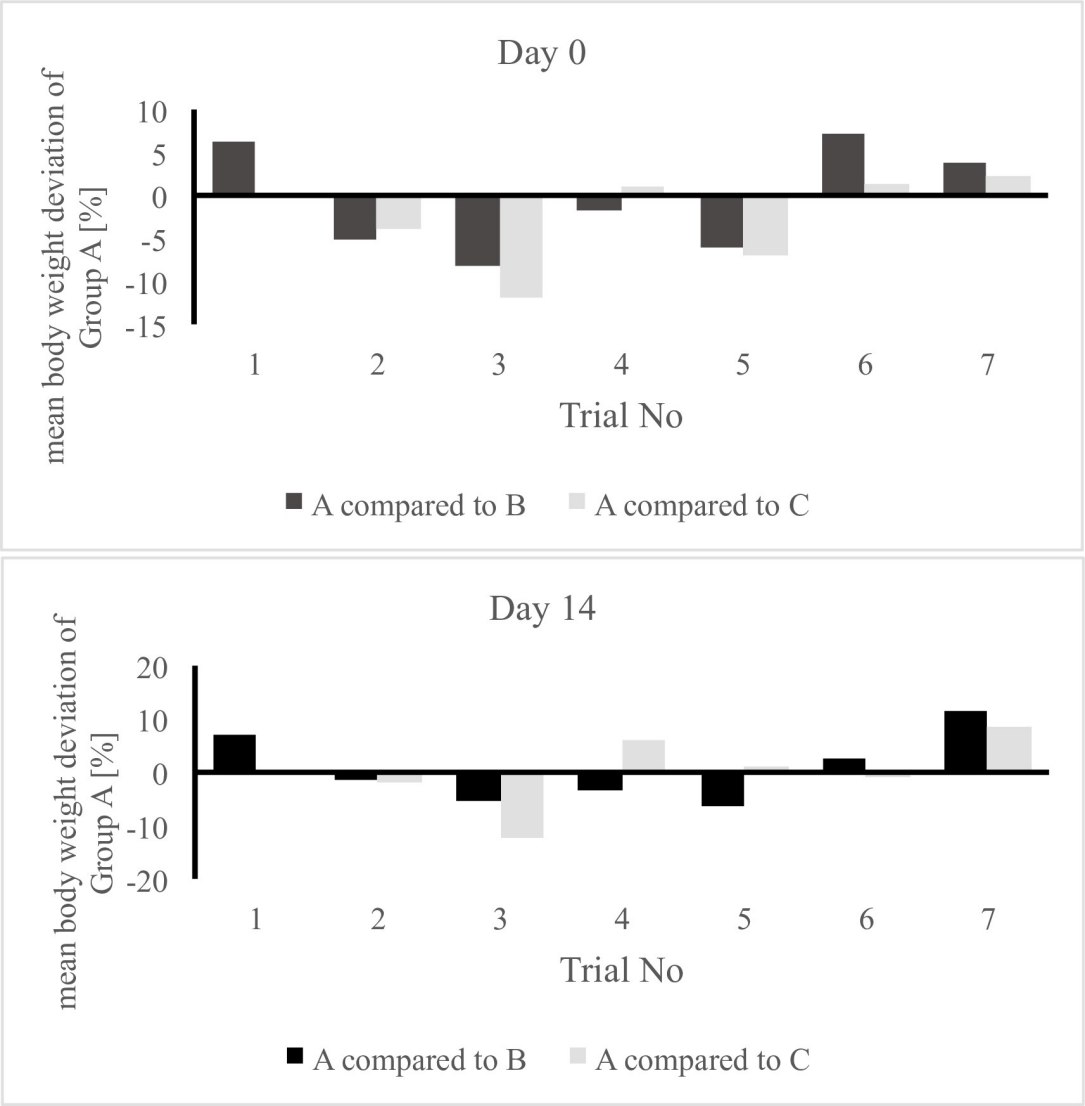
Comparison of the mean body weight (group body weight / no. of individuals) of the contaminated and treated group (A) with the contaminated group (B) and control group (C). The bars indicate the relatively increased or decreased body weight of group A at hatch (day 0) and at the end of the study (day 14).

**Fig 3:**
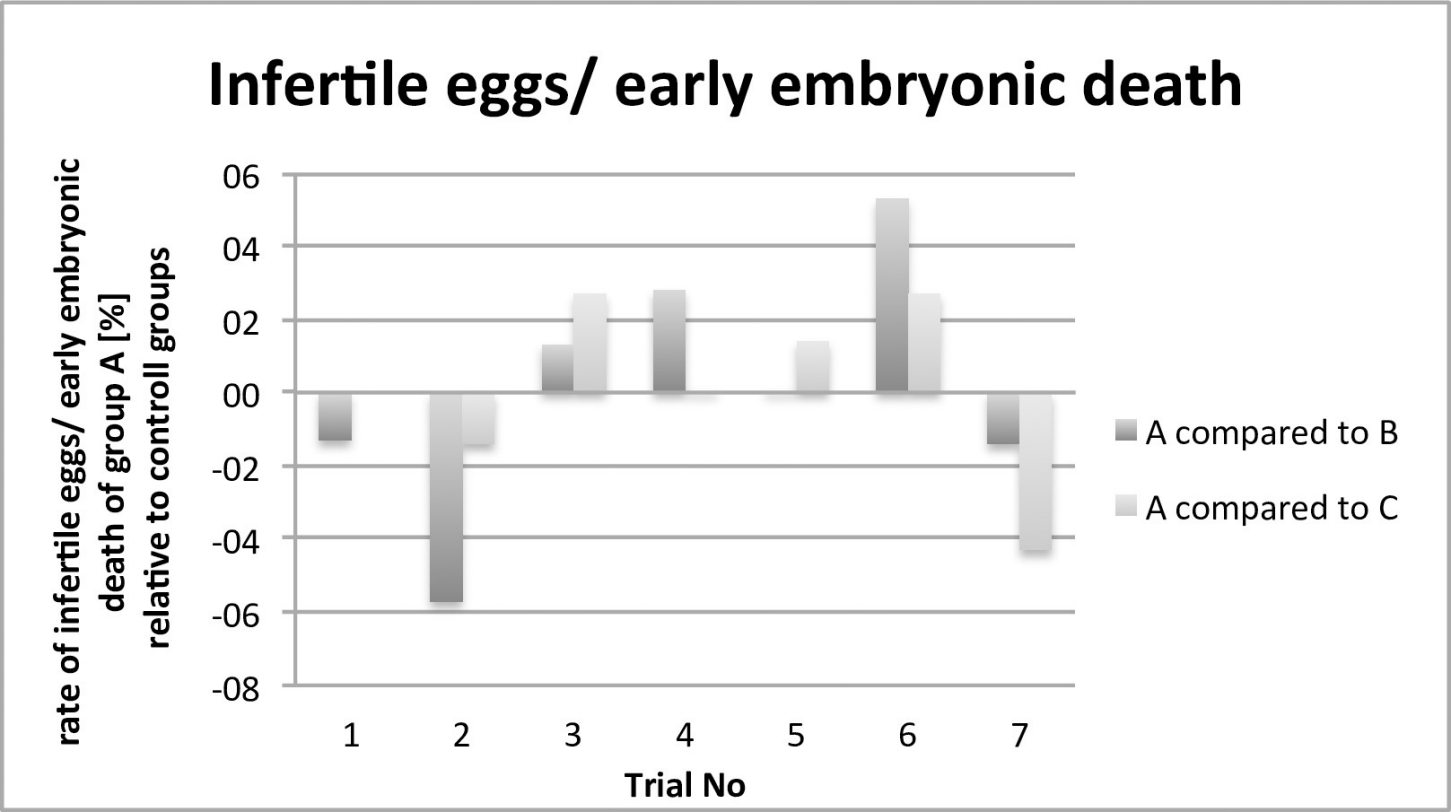
Comparison of the number of infertile eggs/early embryonic death [%] of the contaminated and treated group (A) with the contaminated group (B) and control group (C). The bars indicate the relatively increased or decreased number of infertile eggs/early embryonic death.

**Fig 4:**
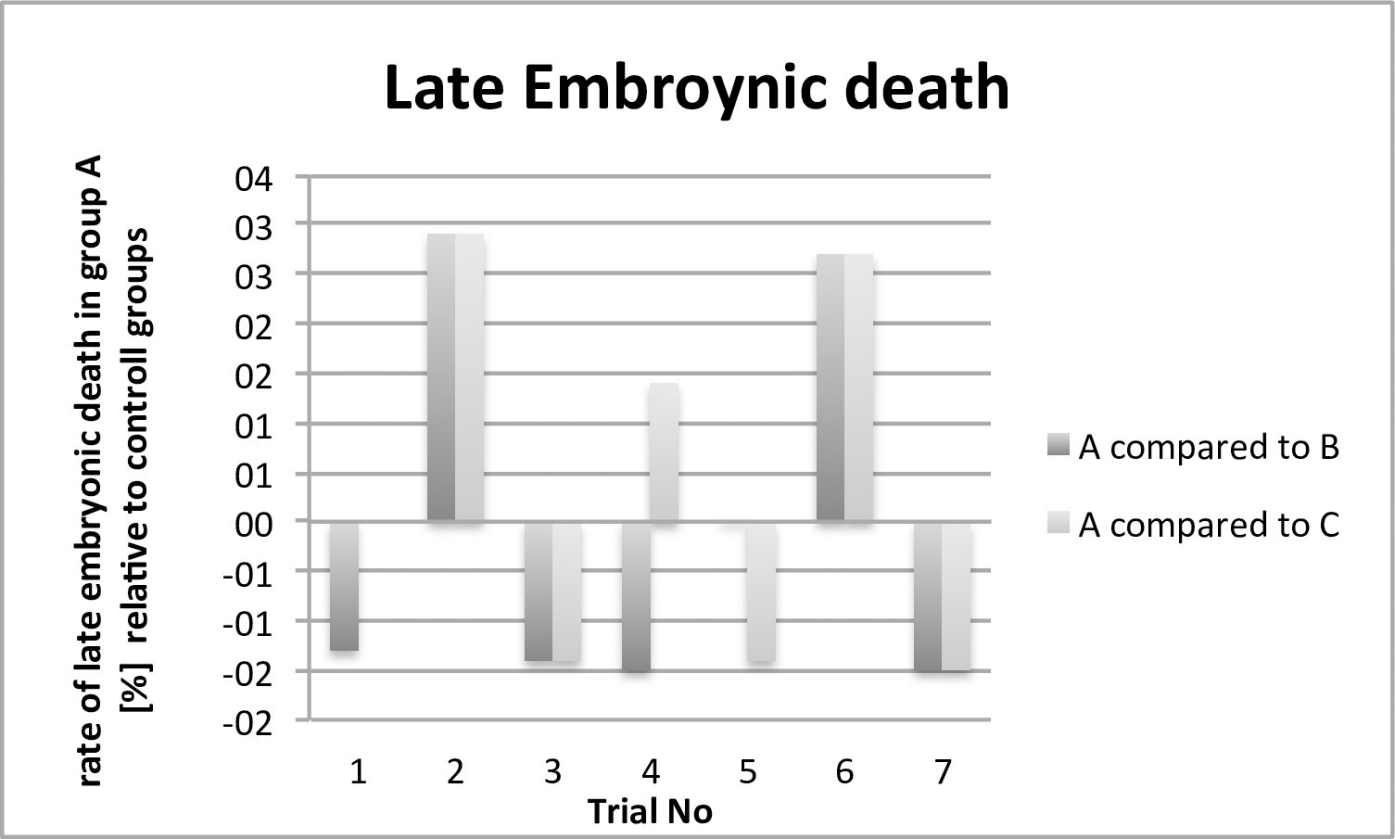
Comparison of the rate of late embryonic death [%] of the contaminated and treated group (A) with the contaminated group (B) and control group (C). The bars indicate the relatively increased or decreased number of late dead embryos.

**Fig 5:**
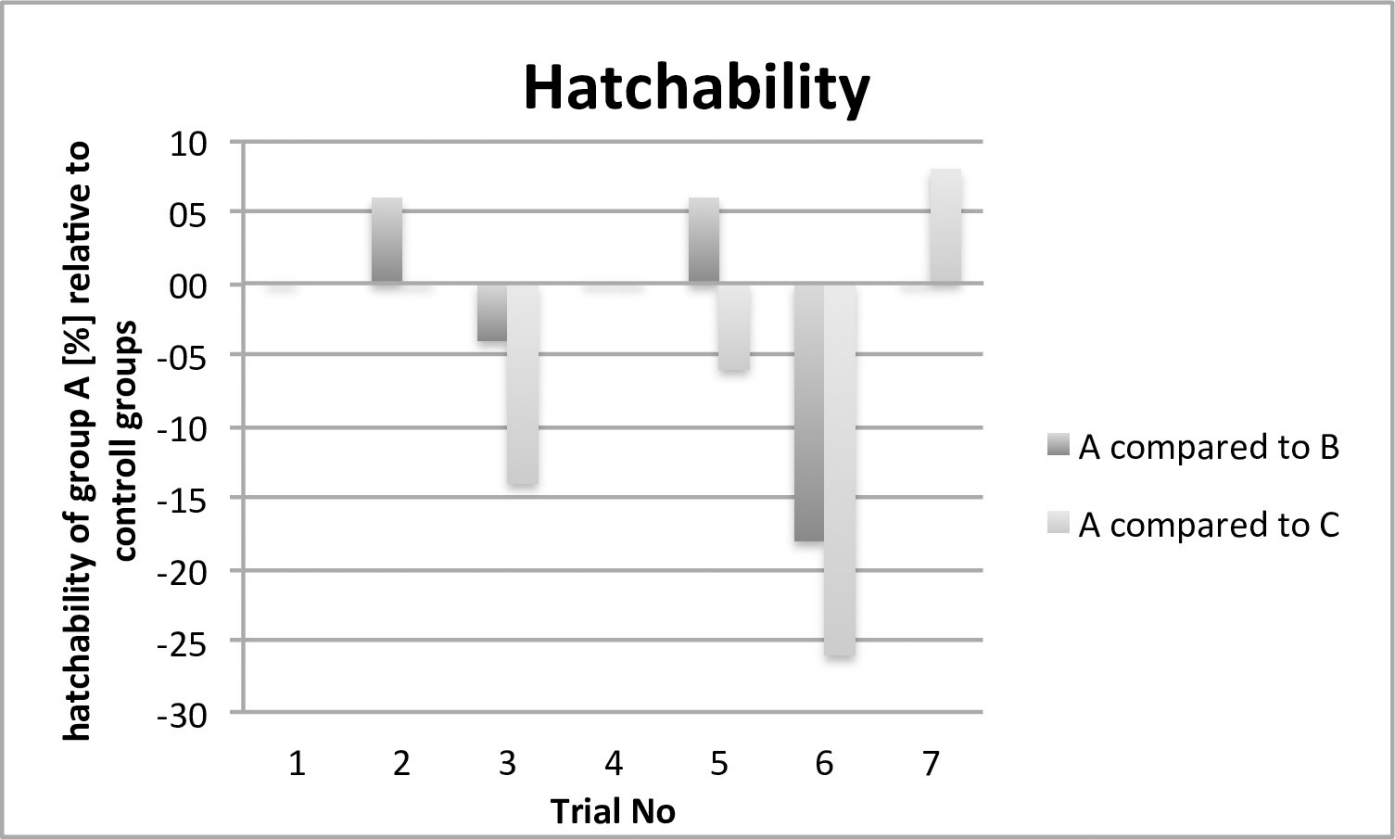
Comparison of the hatchability [%] of the contaminated and treated group (A) with the contaminated group (B) and control group (C). The bars indicate the relatively increased or decreased hatching rate.

**Fig 6:**
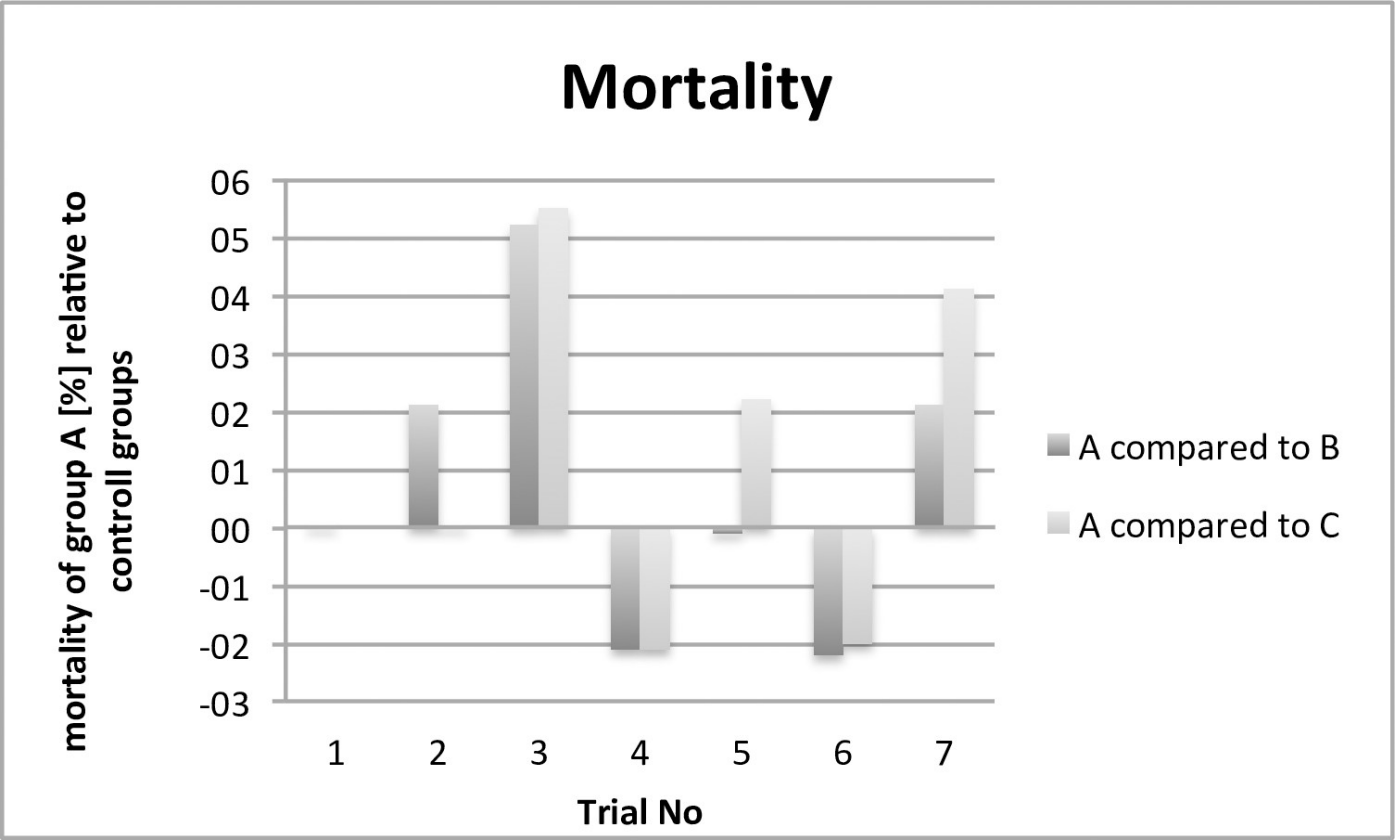
Comparison of the mortality [%] of the contaminated and treated group (A) with the contaminated group (B) and control group (C). The bars indicate the relatively increased or decreased mortality rate.

**Fig 7:**
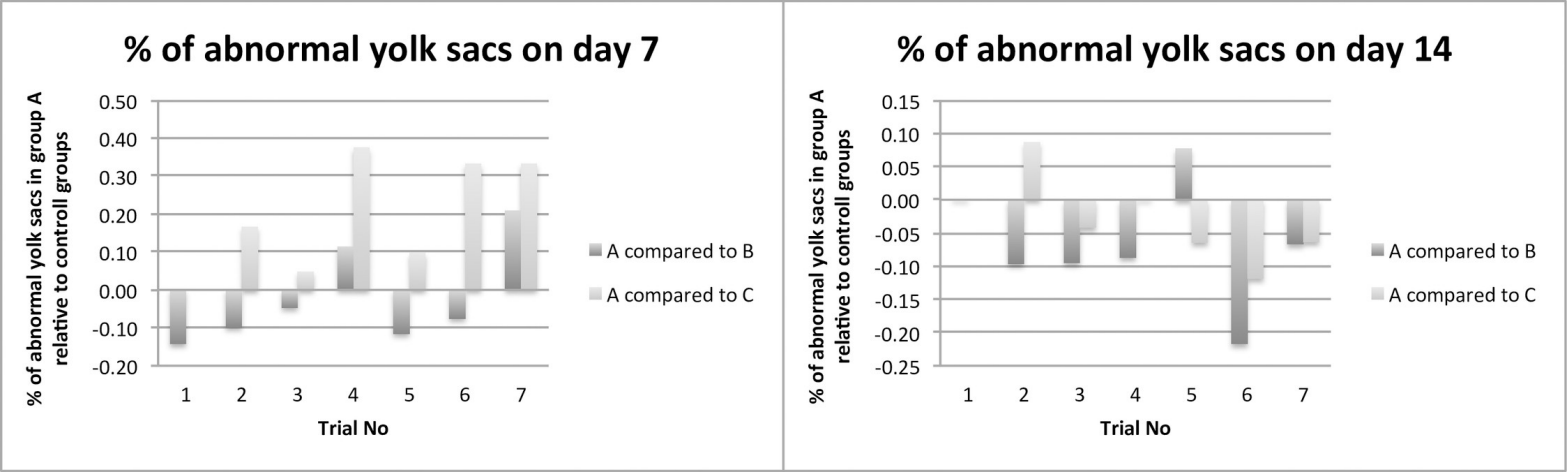
Comparison of the rate of abnormal yolk sacs [%] of the contaminated and treated group (A) with the contaminated group (B) and control group (C). The bars indicate the relatively increased or decreased rate of abnormal yolk sacs on day 7 and 14.

This paper evaluates parameters concerning animal health, results relating to antimicrobial effectivity are part of a different working group and will be published separately. The comparison of the performance and health parameters of group A was of special interest in relation to the contaminated (B) and control group (C). For all trials, the performance parameters body weight development (Fig 2), infertile eggs/early embryonic death (Fig 3), late embryonic death (Fig 4), hatchability (Fig 5) and mortality (Fig 6) are illustrated below. Exemplarily for the recorded health parameters, the occurence of abnormal yolk sacs on day 7 and 14 comparing group A to groups B and C over all trials is displayed in Fig 7. Supplementary data demonstrate the body weight performance for each group in each trial (S1–S7 Figs).

### Trial No. 1—Formaldehyde

Group C was missing due to a technical defect of the incubator. Between groups A and B no significant differences concerning fertility, hatchability and mortality were noted. In group A, one chick was born with three legs and was consequently euthanized. The mean body weight was higher in group A than in group B. During necropsy, more abnormal yolk sacs were found in group B, but the difference was not significant. Histologically, there were no clear differences, only the mean relative liver weight was significantly lower in group A. Because of the missing group C, this trial was repeated (see Trial No. 2). For further information see Table 5.

**Table 5:** Trial 1—Formaldehyde disinfection, breeding and necropsy results.

Group	A: Contaminated, treated		B: Contaminated, untreated		C: Control	
**Infertile eggs/early embryonic death [%]**	4.0		5.3		-	
**Late embryonic death [%]**	2.7		4.0		-	
**Hatchability [%]**	86		86		-	
**Mortality [%]**	0		0		-	
	Day 7 / 14		Day 7 / 14		Day 7 / 14	
**N**	21	22	21	22	-	-
**Mean body weight [g]**	191.0	499.7	185.5	467.1	-	-
**Mean relative liver weight [%]**	5.9	3.9^a^**	5.9	4.5^a^**	-	-
**Mean relative gut weight [%]**	11.3	7.5	11.4	8.0	-	-
**Macroscopy**						
**Abnormal yolk sacs [n]**	2	0	5	0	-	-
**Abnormal kidneys [n]**	0	0	1	0	-	-
**Histology**						
**Liver, extramedullary hematopoiesis [n]**	0	1	3	4	-	-
**Liver, lymphoid follicles [n]**	0	8	0	5	-	-
**Lung, lymphoid follicles [n]**	0	7	2	12	-	-
**Kidneys, lymphoid follicles [n]**	0	3	0	0	-	-

Statistical evaluation conducted only between group A to group B and C

^a, b, c, d^ Superscripts within the row and time indicate significant differences

* indicates p ≤ 0.05 (significant)

** indicates p ≤ 0.01 (highly significant)

#### Trial No. 2 –Formaldehyde

In total, the performance parameters were unremarkable between all groups with nearly identical mean body weights on days 7 and 14. Only group A showed a significantly higher mean relative gut weight than group C on day 7 and a higher mean relative liver weight than group B on day 14. Parameters recorded during necropsy and histologic examination were also unremarkable, the exception being a significantly lower number of lungs with lymphoid follicles in group A compared to groups B and C. Histologically, the livers showed more extramedullary hematopoiesis on day 14 for group A in comparison to group C, but more lymphoid follicles in group C than in group A on the same day. For further information see Table 6.

**Table 6:** Trial 2 –Repeat formaldehyde disinfection, breeding and necropsy results.

Group	A: Contaminated, treated		B: Contaminated, untreated		C: Control	
Infertile eggs/early embryonic death [%]	0		5.7		1.4	
Late embryonic death [%]	2.9		0		0	
Hatchability [%]	96		90		96	
Mortality [%]	2.1		0		2.1	
	Day 7 / 14		Day 7 / 14		Day 7 / 14	
N	24	23	23	22	24	23
Mean body weight [g]	214.3	538.0	231.5	545.4	217.5	549.0
Mean relative liver weight [%]	5.0	5.0^a^*	4.7	3.7^a^*	4.6	3.8
Mean relative gut weight [%]	11.0^a^*	9.0	10.9	9.2	10.5^a^*	9.1
Macroscopy						
Abnormal yolk sacs [n]	7	3	9	5	3	1
Abnormal kidneys [n]	0	0	0	1	0	1
Histology						
Liver, extramedullary hematopoiesis [n]	9	10^a^*	11	10	5	3^a^*
Liver, lymphoid follicles [n]	3	5^a^*	3	9	7	14^a^*
Lung, lymphoid follicles [n]	0^a^**^,^^b^*	9	7^a^**	14	5^b^*	12
Kidneys, lymphoid follicles [n]	2	3	0	3	2	1

Statistical evaluation conducted only between group A to group B and C

^a, b, c, d^ Superscripts within the row and time indicate significant differences

* indicates p ≤ 0.05 (significant)

** indicates p ≤ 0.01 (highly significant)

### Trial No. 3—Hydrogen Peroxide

Hatchability was distinctly reduced in group A compared to group C. Furthermore, mortality was higher in group A than in both control groups. Chicks in group A hatched approximately one day later than those in both control groups. Body weight development in group A was significantly lower than in group C on day 7 and significantly lower than in groups B and C on day 14. In total, group C performed better than both contaminated groups. On day 14, group A had significantly higher mean relative gut weight than groups B and C. The macroscopic examination showed a significantly higher number of abnormal kidneys in group B. Histologically, significant differences appeared to be distributed inconsistently. Group A had a high number of animals with extramedullary hematopoiesis in livers on day 7, while a high number of chicks in group C had lungs with lymphoid follicles on day 14. For further information see Table 7.

**Table 7:** Trial 3 –Hydrogen peroxide disinfection, breeding and necropsy results.

Group	A: Contaminated, treated		B: Contaminated, untreated		C: Control	
Infertile eggs/early embryonic death [%]	4		2.7		1.3	
Late embryonic death [%]	1.3		2.7		2.7	
Hatchability [%]	84		88		98	
Mortality [%]	7.5		2.3		2.0	
	Day 7 / 14		Day 7 / 14		Day 7 / 14	
N =	21	18	21	21	24	24
Mean body weight [g]	179.7^a^**	473.7^b^*^,^^c^**	181.2	500.0^b^*	197.5^a^**	540.3^c^**
Mean relative liver weight [%]	6.2	4.0	6.2	4.0	5.8	4.0
Mean relative gut weight [%]	11.0	8.1^a^*^,^^b^**	10.3	7.6^a^*	11.1	7.6^b^**
Macroscopy						
Abnormal yolk sacs [n]	1	0	2	2	0	1
Abnormal kidneys [n]	0	0^a^*	0	5^a^*	0	0
Histology						
Liver, extramedullary hematopoiesis [n]	8^a^**	5	6	6	1^a^**	6
Liver, lymphoid follicles [n]	2	6	2	2	1	4
Lung, lymphoid follicles [n]	3	1^a^**	2	5	2	11^a^**
Kidneys, lymphoid follicles [n]	0	0	2	0	2	4

Statistical evaluation conducted only between group A to group B and C

^a, b, c, d^ Superscripts within the row and time indicate significant differences

* indicates p ≤ 0.05 (significant)

** indicates p ≤ 0.01 (highly significant)

### Trial No. 4—Low-energy Electron Irradiation

Results were similar between all groups for egg development and hatching rate, however, animals in groups B and C started hatching half a day earlier than those in group A. Mortality was lowest in group A, but the group had a significantly lower body weight than group B on day 7. The difference in body weight is not replicable on day 14. Pathology results demonstrated a lower mean relative liver and gut weight in group C, but no significant differences between the two contaminated groups. Macroscopically, yolk sac retention was found significantly more often in group A in comparison to the negative control group C. Histologically, the incidence of lymphoid follicles in the kidneys was significantly higher in group C than in the other groups. Otherwise, the results were not group-specific. For further information see Table 8.

**Table 8:** Trial 4 –Low-energy electron irradiation disinfection, breeding and necropsy results.

Group	A: Contaminated, treated		B: Contaminated, untreated		C: Control	
Infertile eggs/early embryonic death [%]	7.1		4.3		7.1	
Late embryonic death [%]	1.4		2.9		0	
Hatchability [%]	96		96		96	
Mortality [%]	2.1		4.2		4.2	
	Day 7 / 14		Day 7 / 14		Day 7 / 14	
N	24	23	23	23	23	23
Mean body weight [g]	161.9^a^**	482.6	184.3^a^**	498.6	164.6	455.1
Mean relative liver weight [%]	5.2	4.7^a^**	5.4	4.5	5.4	3.9^a^**
Mean relative gut weight [%]	10.6^a^**	9.5^b^**	10.8	9.4	9.5^a^**	8.6^b^**
Macroscopy						
Abnormal yolk sacs [n]	9^a^**	0	6	2	0^a^**	0
Abnormal kidneys [n]	4	4	0	1	2	0
Histology						
Liver, extramedullary hematopoiesis [n]	2	7	4	4	0	3
Liver, lymphoid follicles [n]	2	13	4	7	4	9
Lung, lymphoid follicles [n]	7	5	5	12	5	7
Kidneys, lymphoid follicles [n]	0^a^*	3	2	1	4^a^*	2

Statistical evaluation conducted only between group A to group B and C

^a, b, c, d^ Superscripts within the row and time indicate significant differences

* indicates p ≤ 0.05 (significant)

** indicates p ≤ 0.01 (highly significant)

### Trial No. 5—Peracetic Acid

In group A, egg development and hatchability were similar to that in both other groups, though hatching started half a day later than in groups B and C. Body weight development was first unremarkable, but then significantly lower than in group B on day 14. For the mean relative liver and gut weights, values in group A were between those in groups B and C. The only significant difference occurring histologically was a higher number of livers with extramedullary hematopoiesis in group B on day 14. For further information see Table 9.

**Table 9:** Trial 5 –Peracetic acid disinfection, breeding and necropsy results.

Group	A: Contaminated, treated		B: Contaminated, untreated		C: Control	
Infertile eggs/early embryonic death [%]	7.1		7.1		5.7	
Late embryonic death [%]	0		0		1.4	
Hatchability [%]	92		86		98	
Mortality [%]	2.2		2.3		0	
	Day 7 / 14		Day 7 / 14		Day 7 / 14	
N	23	22	21	20	25	24
Mean body weight [g]	169.5	420.9^a^*	166.4	448.5^a^*	175.6	416.3
Mean relative liver weight [%]	5.5^a^**	3.9^b^**	5.5	4.3	5.0^a^**	5.0^b^**
Mean relative gut weight [%]	11.7^a^*	8.7^b^**,^c^*	11.9	9.7^b^**	11.6^a^*	7.9^c^*
Macroscopy						
Abnormal yolk sacs [n]	5	5	7	3	3	7
Abnormal kidneys [n]	1	3	0	0	2	1
Histology						
Liver, extramedullary hematopoiesis [n]	6	2^a^**	5	12^a^**	2	2
Liver, lymphoid follicles [n]	3	7	3	10	3	3
Lung, lymphoid follicles [n]	1	11	3	5	5	9
Kidneys, lymphoid follicles [n]	0	2	2	5	1	3

Statistical evaluation conducted only between group A to group B and C

^a, b, c, d^ Superscripts within the row and time indicate significant differences

* indicates p ≤ 0.05 (significant)

** indicates p ≤ 0.01 (highly significant)

### Trial No. 6—Essential Oils–Spray Application

Group A showed a remarkably low hatching rate, but also higher number of infertile and dead eggs in comparison to groups B and C. In group C, two chicks were euthanized due to congenital deformation (cross beak and four-legged). Apart from that, mortality and body weight development appeared to be relatively unremarkable. At necropsy, significantly different mean relative gut weights were recorded between group A and both other groups. Apart from that, the number of remarkable yolk sacs was significantly higher for group A in comparison to group C, but lower than in group B. For further information see Table 10.

**Table 10:** Trial 6 –Essential oils spray disinfection, breeding and necropsy results.

Group	A: Contaminated, treated		B: Contaminated, untreated		C: Control	
Infertile eggs/ early embryonic death [%]	8		2.7		5.3	
Late embryonic death [%]	4		1.3		1.3	
Hatchability [%]	72		90		98	
Mortality [%]	0		2.2		2.0	
	Day 7 / 14		Day 7 / 14		Day 7 / 14	
N	18	18	22	22	23	23
Mean body weight [g]	174.9	448.1	183.6	436.4	168.9	452.0
Mean relative liver weight [%]	6.0	4.1	5.8	4.4	5.5	4.3
Mean relative gut weight [%]	10.9	8.7^a^**^,^^b^**	11.0	6.5^a^**	11.2	7.6^b^**
Macroscopy						
Abnormal yolk sacs [n]	6^a^**	1	9	6	0^a^**	4
Abnormal kidneys [n]	2	1	1	2	3	0
Histology						
Liver, extramedullary hematopoiesis [n]	2	9	1	7	1	5
Liver, lymphoid follicles [n]	2	8	2	6	2	5
Lung, lymphoid follicles [n]	4	3	3	5	5	10
Kidneys, lymphoid follicles [n]	3	3	0	5	0	3

Statistical evaluation conducted only between group A to group B and C

^a, b, c, d^ Superscripts within the row and time indicate significant differences

* indicates p ≤ 0.05 (significant)

** indicates p ≤ 0.01 (highly significant)

### Trial No. 7—Essential Oils–Fogging

Egg development and hatchability were unremarkable. Mortality was slightly higher for group A than for groups B and C. Body weight was significantly lower in group A than in group B on day 7, despite starting off at the same weight on day 1. On day 14, group A turned out to be significantly heavier than both control groups. Mean relative liver weights in group A were significantly lower than in group B on day 14. Mean relative gut weights were higher than in both control groups on day 7, but were between those found in groups B and C on day 14. On day 7, significantly more retained yolk sacs were found in animals in group A than in group C. The number of retained yolk sacs increased in all groups by day 14, but there were no significant differences between the groups. Significant differences in number of animals with extramedullary hematopoiesis and lymphoid follicles were noticed between groups A and B. For further information see Table 11.

**Table 11:** Trial 7 –Essential oils ultrafogger disinfection, breeding and necropsy results.

Group	A: Contaminated, treated		B: Contaminated, untreated		C: Control	
Infertile eggs/early embryonic death [%]	0		1.4		4.3	
Late embryonic death [%]	1.4		2.9		2.9	
Hatchability [%]	98		98		90	
Mortality [%]	4.1		2.0		0	
	Day 7 / 14		Day 7 / 14		Day 7 / 14	
N	24	23	24	24	23	22
Mean body weight [g]	156.0^a^**	512.3^b^**^,^^c^*	185.4^a^**	460.0^b^**	168.9	471.7^c^*
Mean relative liver weight [%]	5.3	3.9^a^*	5.5	4.1^a^*	5.4	4.1
Mean relative gut weight [%]	12.9^a^**^,^^b^**	9.1^c^**^,^^d^**	10.7^a^**	8.4^c^**	11.8^b^**	9.7^d^**
Macroscopy						
Abnormal yolk sacs **[n]**	8^a^**	9	3	11	0^a^**	10
Abnormal kidneys **[n]**	1	0	1	0	0	0
Histology						
Liver, extramedullary hematopoiesis **[n]**	4	8^a^*	6	2	3	3^a^*
Liver, lymphoid follicles [n]	5	5^a^*	5	14^a^*	7	10
Lung, lymphoid follicles [n]	0	16	3	18	2	17
Kidneys, lymphoid follicles [n]	2	4	2	4	0	3

Statistical evaluation conducted only between group A to group B and C

^a, b, c, d^ Superscripts within the row and time indicate significant differences

* indicates p ≤ 0.05 (significant)

** indicates p ≤ 0.01 (highly significant)

## Discussion

The aim of this study was to evaluate different egg disinfection procedures on ESBL-producing *E*. *coli* contaminated hatching eggs. ESBL-producing *E*. *coli* in poultry is reported to have varying pathogenicity.[36] The ESBL-producing *E*. *coli* used in this study was isolated from one day old chicks at broiler farms.[39] No special information was available concerning the health of the chicks. For this study, pathological changes were not expected. In all trials, the hatched chicks were clinically healthy, apart from some solitary cases in which individuals had to be euthanized or died spontaneously. These chicks were necropsied, but the results were not included into the statistical evaluation since they were non-standardized samples. Statistical evaluation was conducted between all three groups of each separate trial, the number of chicks per group was adequate for these purposes. By comparing the contaminated groups A and B, the impact of the disinfectant onto group A was evaluated by comparing the contaminated groups A and B, since this was the only independent variable between these groups. Comparisons of groups B and C evaluated the effects of the artificial contamination on group B (and by extension, on group A). The clinical findings indicate that the experimental contamination with ESBL producing *E*. *coli* appeared to have a different impact in the different trials, although the same ESBL producing *E*. *coli* strain was used in each trial. When comparing groups A and C, both contamination and disinfection were independent variables. Results were considered relevant, when group A performed worse, in terms of lower hatchability, higher mortality or decreased body mass development, than groups B and C, leaving the disinfectant as the influencing variable. Significances between groups B and C were not included in the interpretation, as they were not relevant to the study aim.

The body weight development was recorded, but divergence due to different hatching times was possible. In some cases the time frame in which the chicks hatched differed by more than 12 hours. This was recorded and considered during interpretation. Additionally, the chicks were provided with food up to their euthanasia so there was a period of about four hours between the necropsies of group A and C, where chicks in group A could still feed. Food withdrawal was not considered since this would probably have had a significant influence on the intestinal content and the body weight, as well. The relatively increased or decreased body weight of the animals in group A in comparison to those in groups B and C shows however, that the tendencies were consistent from day 0 to day 14, even though the degree of difference varied.

As one part of different steps for the assessment of the body health and impact of the egg contamination and the disinfection method used, several parameters were registered during necropsy. We recorded the mean relative liver and gut weight presuming that liver and gut weights increase if there is an infectious challenge to these organs. Hepatomegaly, for instance, is a common sign of diseases associated with a variety of etiologies. There were no consistent variations in these parameters that would have indicated significant effects of specific treatments. Group C tended to have the lightest livers, but there seemed to be multiple influences on the liver weight, making interpretation of these results impossible. Gut weights mostly showed no tendency towards one group. Macroscopically, the presence of yolk sacs in 7- and 14-day old chicks is a parameter for impaired chick development.[50, 51] During necropsy, we differentiated between yolk sac retention and yolk sac inflammation, but the number of inflamed yolk sacs was low, so that only differences in the number of normal and abnormal yolk sacs were evaluated statistically, thus combining the afore mentioned categories. The distribution of abnormal yolk sacs showed a tendency towards the ESBL producing *E*. *coli* contaminated groups A and B. Deviations of texture and colour of the kidneys were noted, but appeared to be distributed inconsistently between the groups, indicating that the findings were of an unspecific nature.

Since the animals in the study were not expected to develop severe clinical signs of disease following ESBL-producing *E*. *coli* egg contaminations, the histologic examination was planned to provide information on the general health and infection status of the chicks, providing an indication whether the immune system was more challenged in some groups than in others. A noticeably challenged immune system, e. g. in the form of lymphoid follicles, could be a consequence of the ESBL-producing *E*. *coli* contamination, but also of contact with other (bacterial or viral) infectious agents in the environment. Furthermore, the disinfection method itself should also be considered as having a positive or negative impact on the organ function. In this context, the occurrence of hematopoiesis in the liver was especially interpreted as host response to an unspecified challenge. This parameter appeared to be influenced by multiple factors and did not show a specific tendency. Lymphoid follicles in tissues were considered as a sign of an active immune reaction, but without further identification of the cause. In general, lymphoid follicles in lung and liver seemed to increase with age, so environmental influences might also have played a role.

Summarizing, the necropsy results did not allow to draw specific conclusions with regard to an immune reaction following infection or disinfection, as the results were rather vague and probably were the result of a general immunologic challenge depending on the general infectious load. This is conceivable as *E*.*coli* can regularly be isolated from healthy chicken and the ESBL strain used originated from a healthy chicken flock. It can however be concluded that the pathology results did not demonstrate any toxic effect that might be caused by the disinfection methods tested.

### Trials No. 1 and 2 –Formaldehyde

As control group C was missing, the first trial was repeated. The repeated trial had good results concerning chick performance and animal health. The reported negative effects of formaldehyde on the respiratory tract of chicks [4, 5] were not replicable, most likely because fumigation was conducted before incubation and not around the time of hatching. For instance, we found less lymphoid follicles in lungs and livers in the disinfected group than in both control groups, so at least the tissues were not damaged to an extent that predisposed the animals for secondary infections.

### Trial No. 3—Hydrogen Peroxide

In this trial, it was remarkable, that both contaminated groups performed worse than control group C regarding e.g. hatchability and body weight development, with group A showing even worse results than group B on day 14. One possible explanation for the lower body weight development in group A might be a delayed onset of the hatching process. On the other hand, reasons for the delayed hatching of animals in this group were not known. Necropsy results were similar for animals in groups A and B and differences to group C were inconsistent. Perhaps the artificial infection with ESBL producing *E*. *coli* had a higher impact than in the other trials. The development of the body weight of the animals in group A indicated a negative effect of the disinfectant, which is surprising given the number of published studies reporting positive experiences with this disinfectant. We see a need to gather further information in field trials.

### Trial No. 4—Low-energy Electron Irradiation

This trial showed overall homogenous results between all groups. Necropsy still revealed macroscopic differences between the contaminated groups and the negative control group C that might be a result of the experimental ESBL producing *E*. *coli* infection. The use of radiation on hatching eggs has only been reported once [19], however its application in the food industry, as well as its effectivity against *E*. *coli* [59, 60] has been reported. On the other hand, higher radiation doses have been shown to have a negative impact on live organisms was apparent, e.g. on the sprouting of seeds [61, 62]. We did not observe any mutagenic effect or pathologically changed organs in the animals in our trial, so this new method met the safety requirements that were imposed in this study. So far, the use of irradiation has been tested only once on hatching eggs [19] with inconspicuous results in the test groups. Other types of irradiation, like gamma-radiation [63] or X-ray [64], have been tested on hatching eggs with varying results ranging from improved to reduced hatching rates. Especially in regard to microbial resistance to chemical disinfectants [65, 66], a different disinfection approach is welcome. However, *E*. *coli* have reportedly developed resistance mechanisms against radiation effects as well [67, 68].

### Trial No. 5—Peracetic Acid

This experimental trial showed no pronounced negative differences between group A and the control groups. When using peracetic acid, caution should be exercised as cases of reactive airway dysfunction syndrome and asthma have been reported. Asthma occurred when peracetic acid and hydrogen peroxide were used in combination [69]. In our trial however, no effects of this sort were to be expected as we disinfected egg shells and did not expose respiratory tissue to the disinfectant.

### Trial No. 6—Essential oils–Spray Application

Hatchability in group A was clearly reduced, but an elevated number of infertile eggs / eggs with early embryonic death was also apparent. After hatching, the performance and health of the chicks in group A did not differ significantly from the control groups. The disinfectant, however, was deemed unsuitable to be tested in the field trial. A probable explanation for the reduced hatching rate is that the oily disinfectant consistency led to occlusion of the eggshell pores which caused a reduced loss of moisture and reduced supply of oxygen for the chicken embryos. Similar observations have been made with table eggs [70, 71], where oily substances were used to prevent loss of moisture, thus keeping the eggs fresh for a longer time. After consulting with the disinfectant producer, the trial was repeated using a different method of application (see Trial 7).

### Trial No. 7 -Essential oils–Fogging

In total, good results concerning chick performance and animal health were achieved. There was a higher mortality in group A, but in the range of +/-1 animal, which was considered as no effect. When using essential oils, the method of application seems to have a high impact on fertility and hatchability. In this trial, the number of retained yolk sacs in all groups was high on day 14, when usually the number was usually lower than on day 7. This implies that the chicks had problems with their metabolic processes and could not absorb yolk sac nutrients. Since all groups were affected, neither the disinfection nor the ESBL producing *E*. *coli* contamination are likely to have been decisive factors.

### Limitations

We endeavored to provide identical settings for all groups and trials, but had to house the different groups in different rooms due to animal health regulations that apply when working with pathogens. Though facility equipment, e.g. air supply and heating, was the same, we did measure slightly different climatic conditions using a continuous thermometer and hygroscope. The same effort was made during the incubation period, using three separate incubators of the same model that were programmed to identical settings. There were measurable differences with regard to temperature and humidity, as the control systems do not work as accurately as in commercial hatcheries. Also, as this was considered a preliminary study, no replications were conducted for each disinfectant trial. This was always taken into account when evaluating the feasibility of statistical evaluations.

Concerning fertility, eggs that appeared empty on candling were not opened. It was therefore not possible to differentiate between infertile eggs and early embryonic death up to the first 24 hours. This might be considered a critical aspect, as fertility is determined in the mother hen, whereas early embryonic death might already be related to the use of hatching egg disinfectants. However, this was compensated by comparison with the reference groups, which originated from the same parent flocks, thus were expected to have the identical egg fertility rate.

### Conclusion

All disinfectants except for the essential oils preparation as spray application were considered to have no negative effect on performance and health of the chicks. Only certain limiting aspects concerning the body weight development were seen with hydrogen peroxide. The tested disinfection protocols have been used in subsequent field trials to record data on a larger number of animals under field conditions.

## Supporting information

S1 Fig Graphic illustration of the body weight development [g] of groups A, B and C in Trial no 1 over the period of 14 days. (TIF)

S2 Fig Graphic illustration of the body weight development [g] of groups A, B and C in Trial no 2 over the period of 14 days. (TIF)

S3 Fig Graphic illustration of the body weight development [g] of groups A, B and C in Trial no 3 over the period of 14 days. (TIF)

S4 Fig Graphic illustration of the body weight development [g] of groups A, B and C in Trial no 4 over the period of 14 days. (TIF)

S5 Fig Graphic illustration of the body weight development [g] of groups A, B and C in Trial no 5 over the period of 14 days. (TIF)

S6 Fig Graphic illustration of the body weight development [g] of groups A, B and C in Trial no 6 over the period of 14 days. (TIF)

S7 Fig Graphic illustration of the body weight development [g] of groups A, B and C in Trial no 7 over the period of 14 days. (TIF)
