# Supplementary figures and images for: Preliminary study: Health and performance assessment in broiler chicks following application of six different hatching egg disinfection protocols
Source: PLoS One. 2020 May 14;15(5):e0232825. doi: 10.1371/journal.pone.0232825 (PMC7224537; doi:10.1371/journal.pone.0232825)

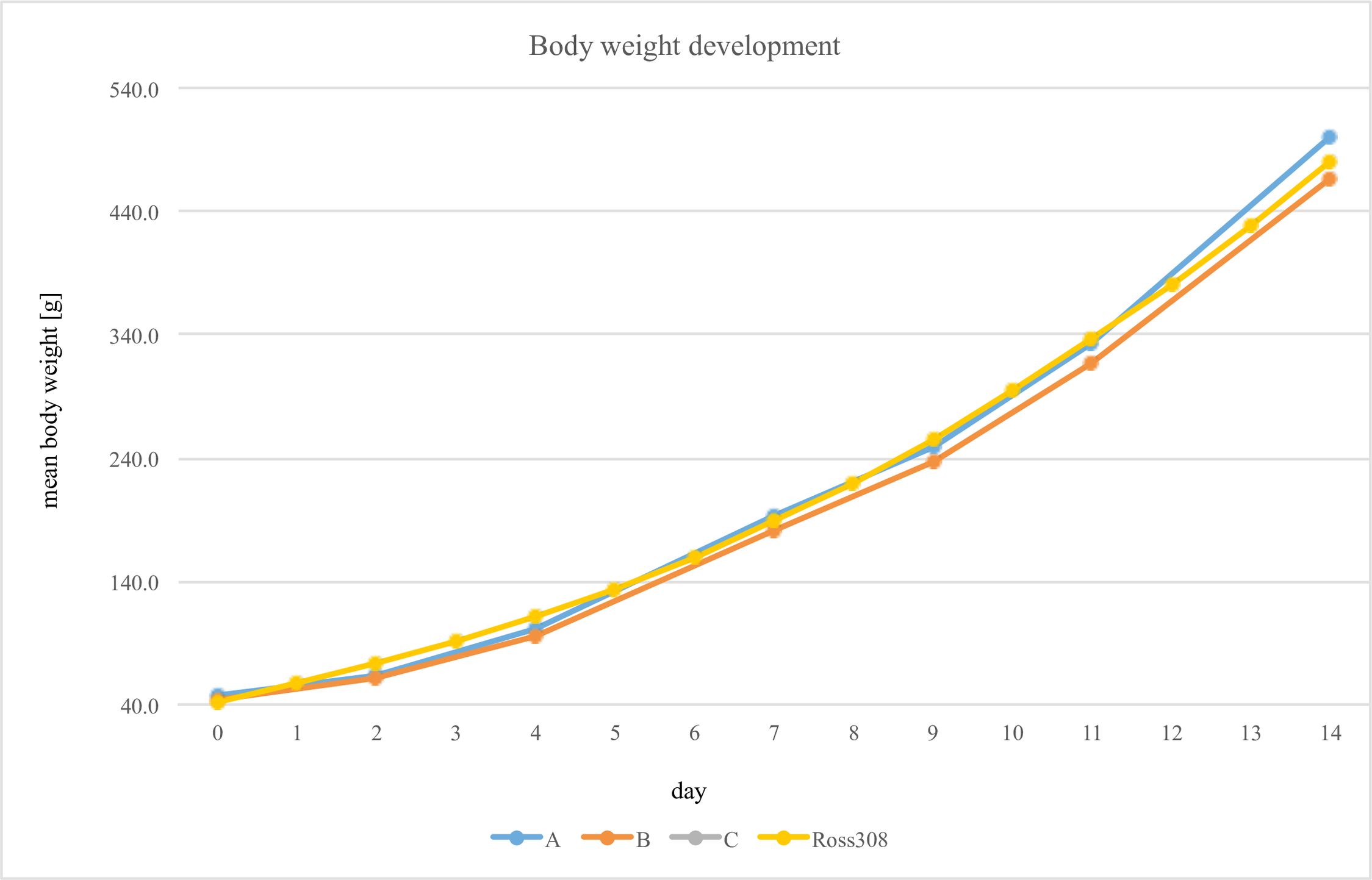

Supplement: S1 Fig — (TIF) [file pone.0232825.s001.tif]

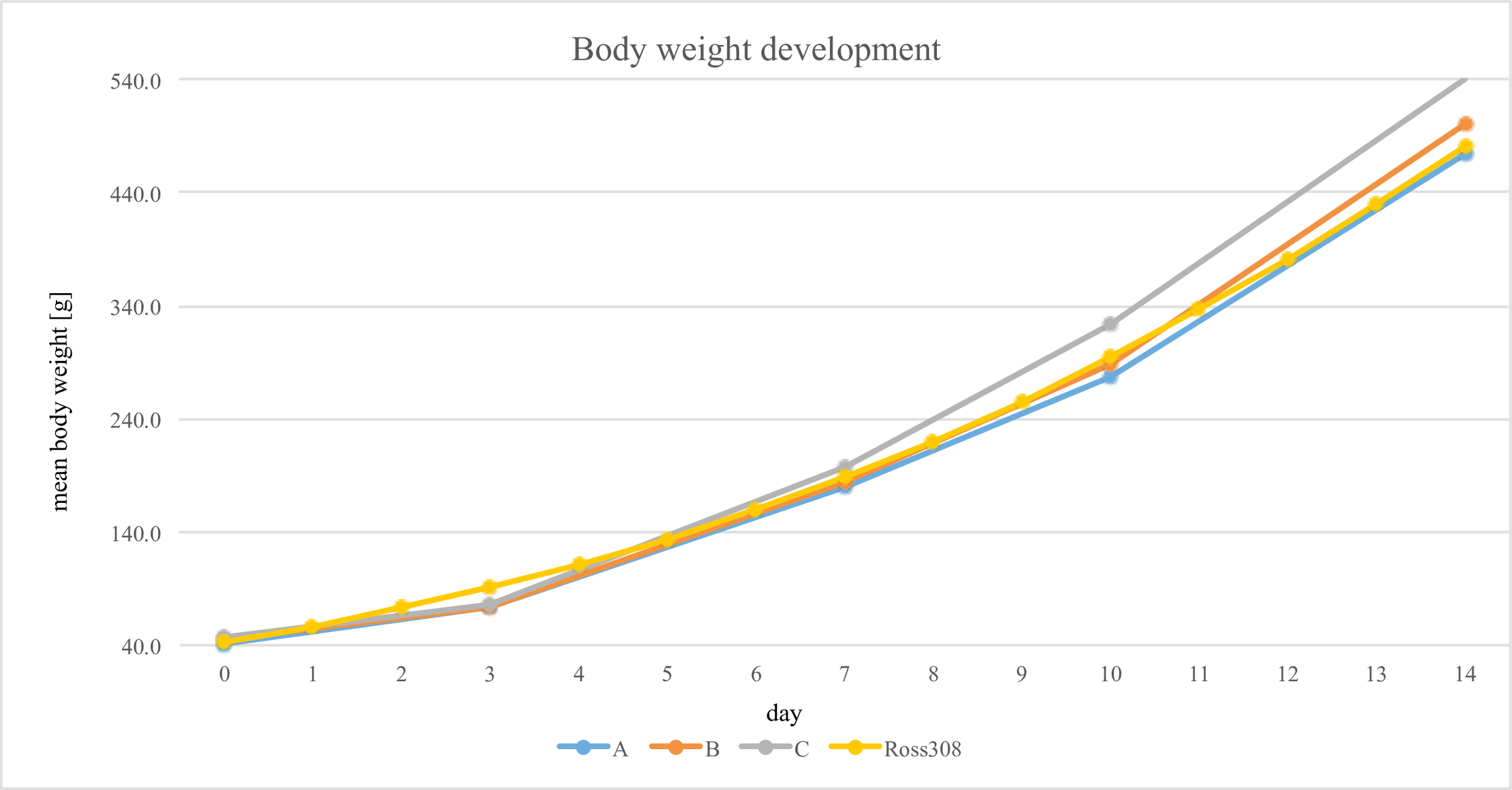

Supplement: S2 Fig — (TIF) [file pone.0232825.s002.tif]

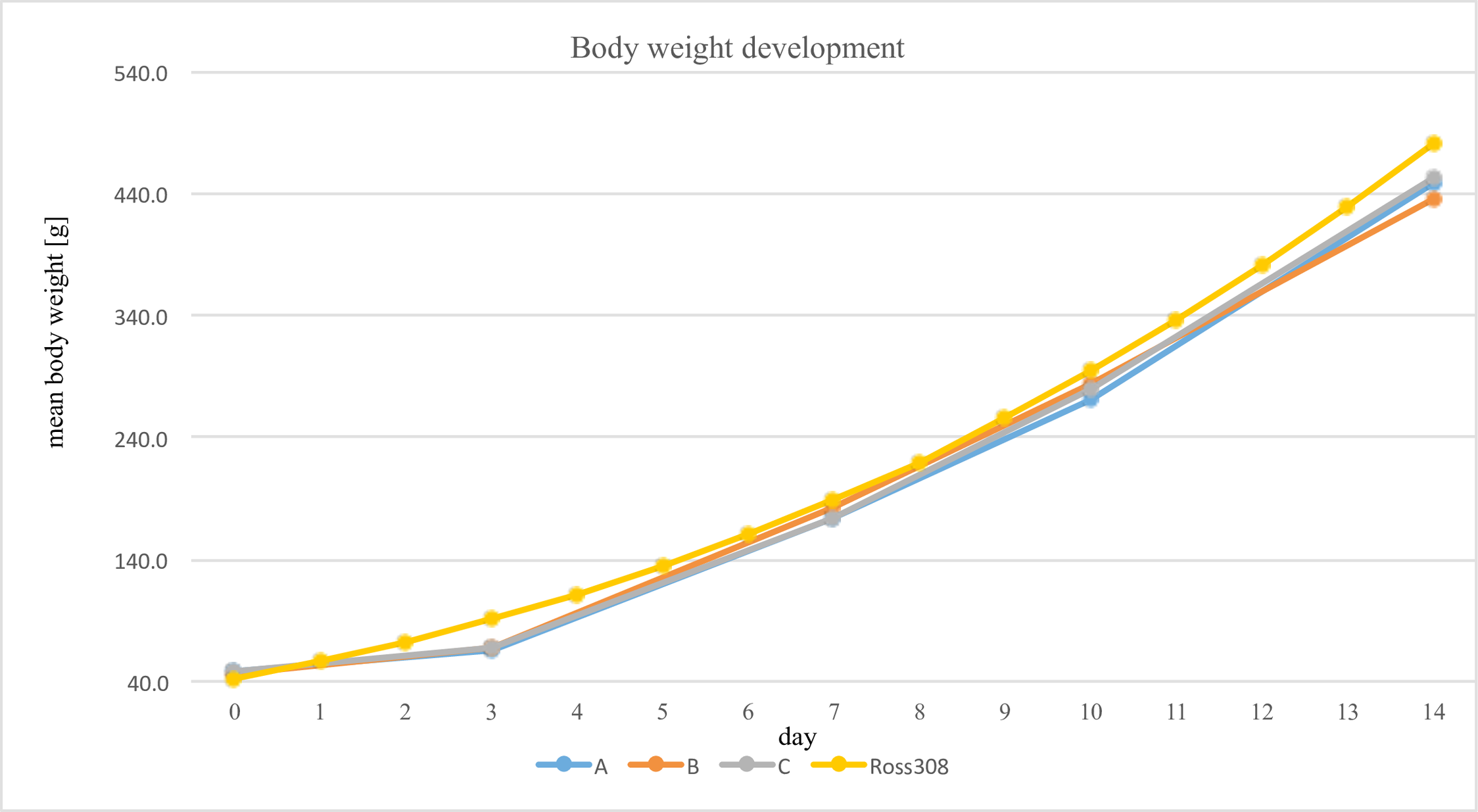

Supplement: S3 Fig — (TIF) [file pone.0232825.s003.tif]

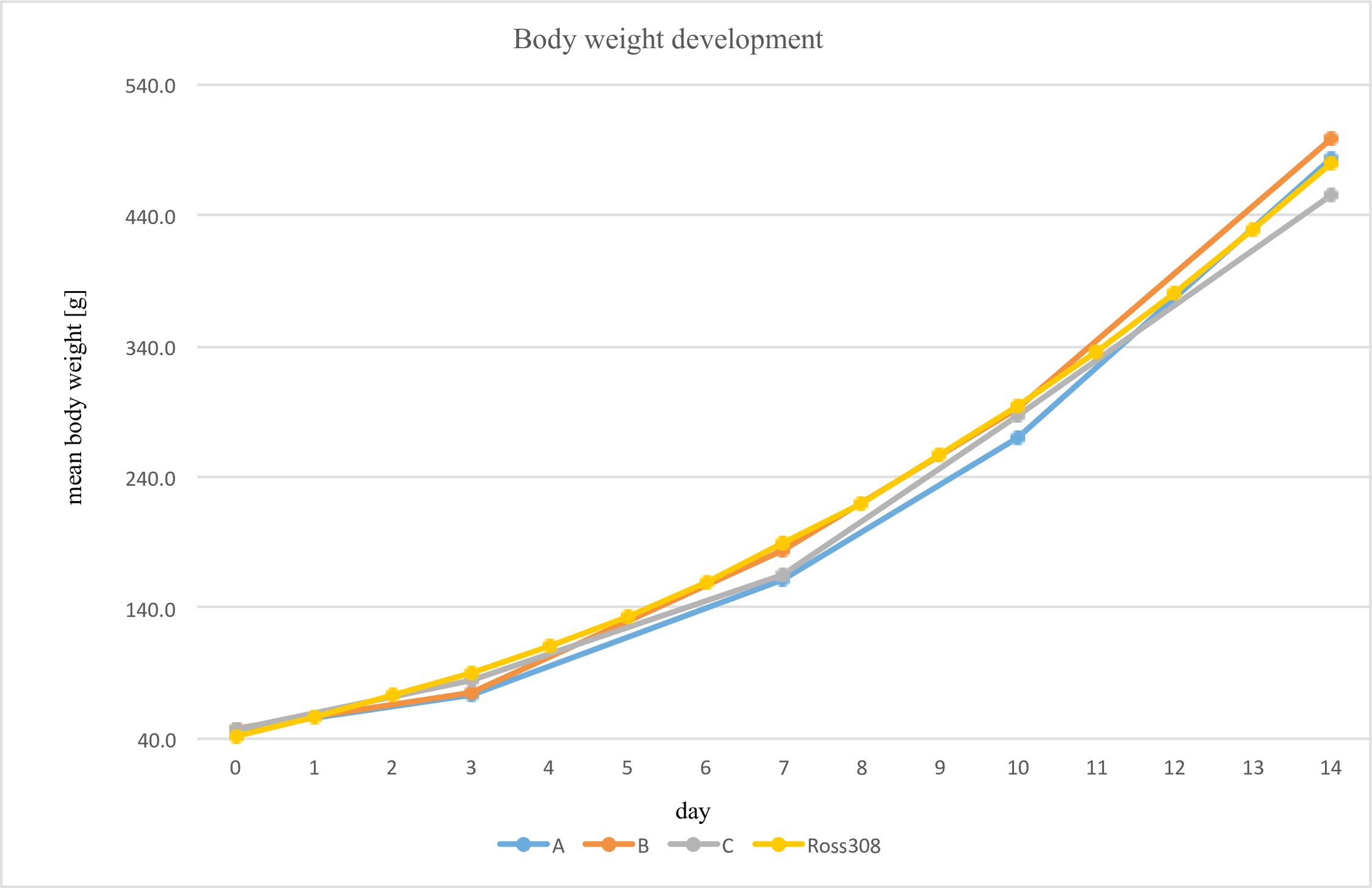

Supplement: S4 Fig — (TIF) [file pone.0232825.s004.tif]

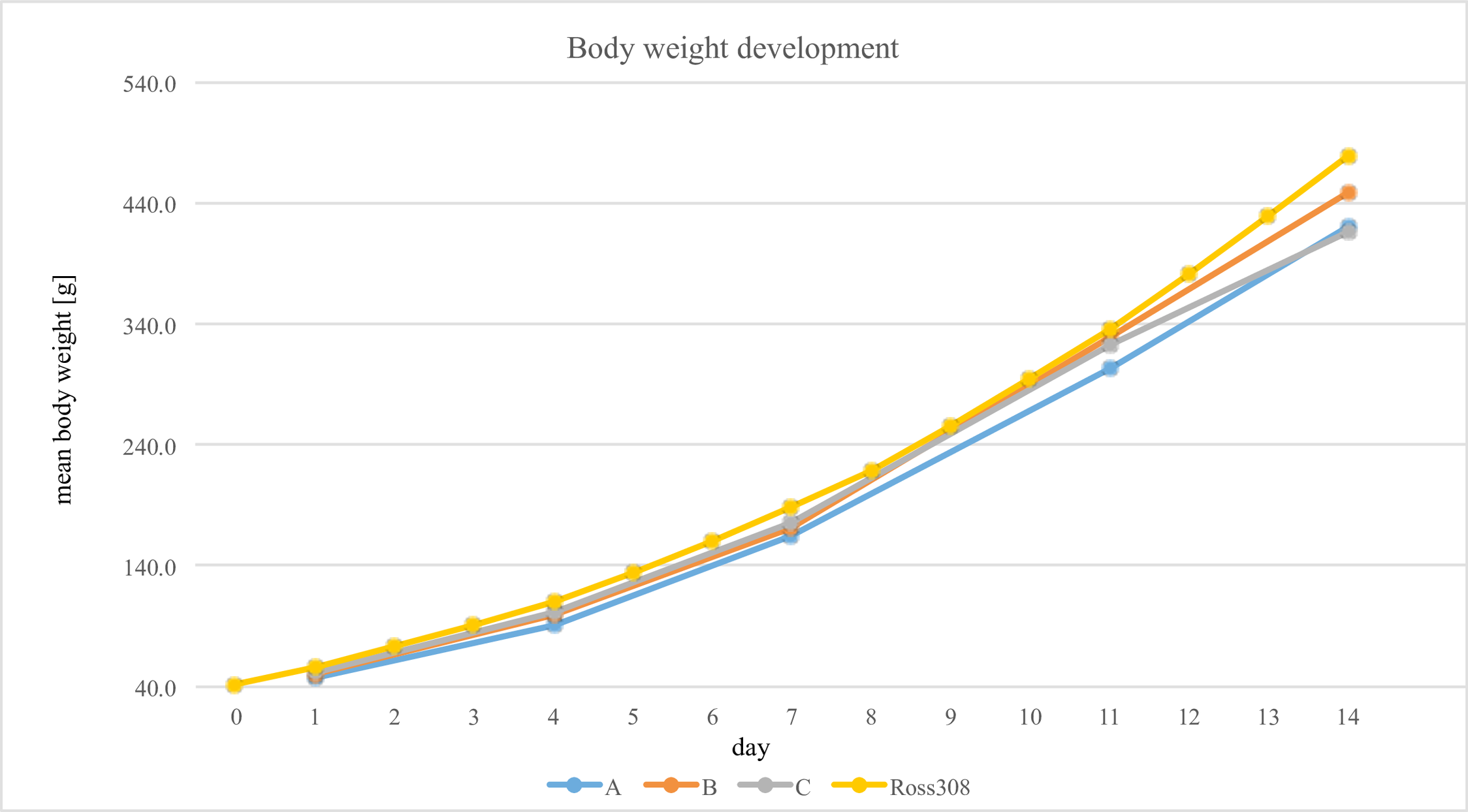

Supplement: S5 Fig — (TIF) [file pone.0232825.s005.tif]

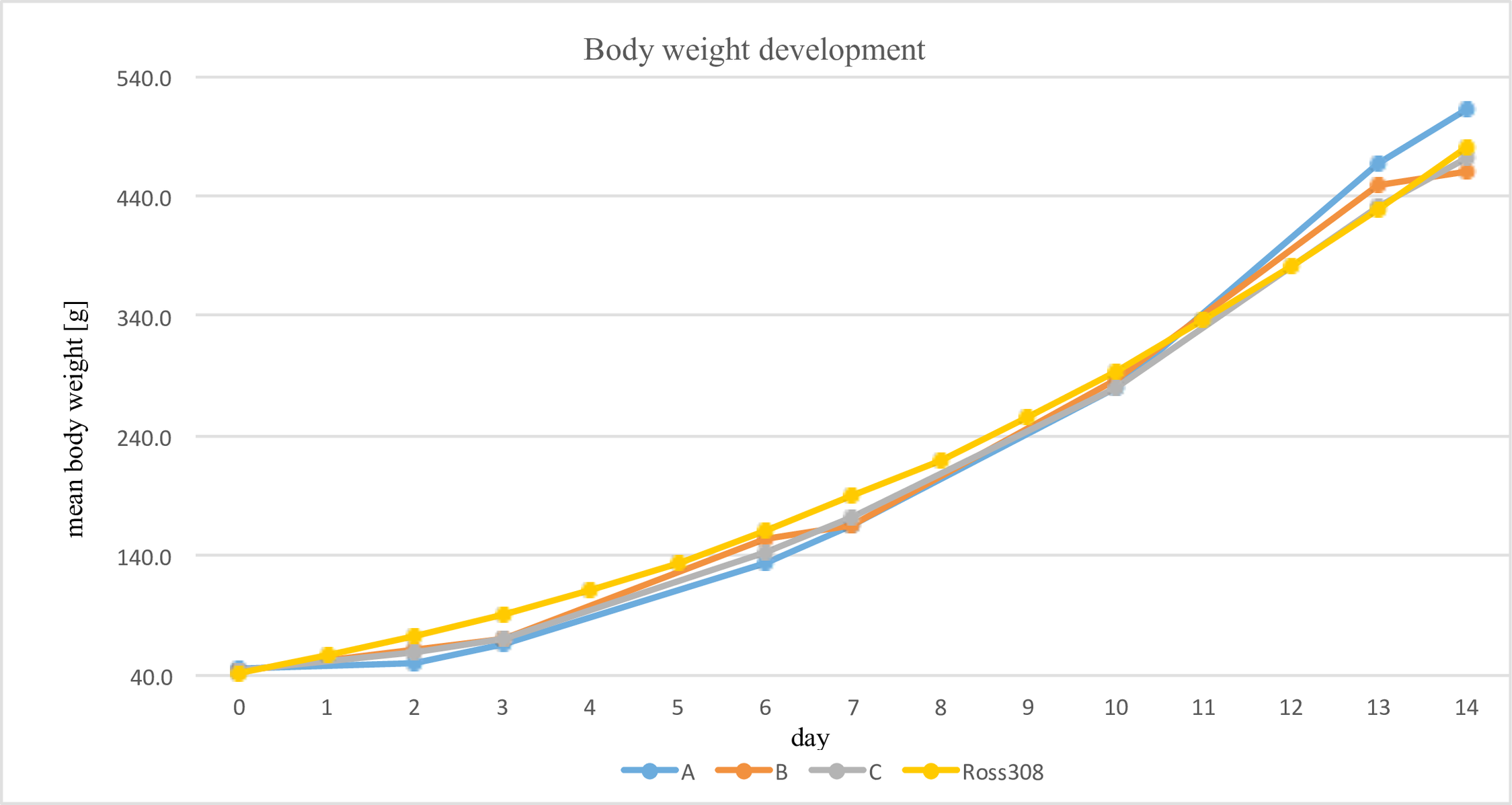

Supplement: S6 Fig — (TIF) [file pone.0232825.s006.tif]

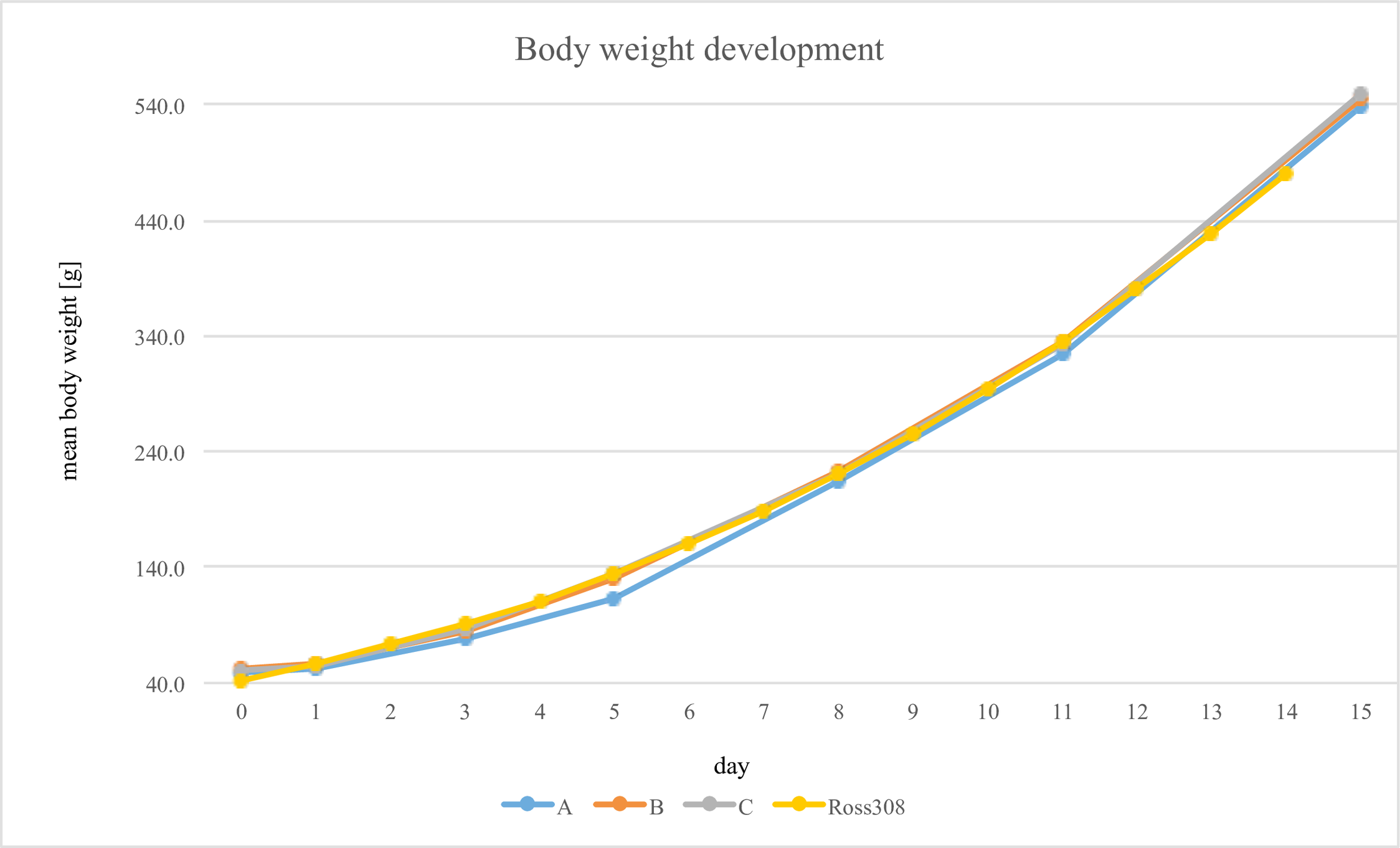

Supplement: S7 Fig — (TIF) [file pone.0232825.s007.tif]
